# The utility of automated artificial intelligence‐assisted digital cytomorphology for bone marrow analysis in diagnostic haemato‐oncology

**DOI:** 10.1002/ctm2.70364

**Published:** 2025-07-09

**Authors:** David Starostka, Richard Dolezilek, Hans Michael Kvasnicka, Milos Kudelka, Petra Miczkova, Eva Kriegova, David Kolacek, Barbora Sotkovska, Tomas Anlauf, Jarmila Juranova, Katerina Chasakova, Sona Kolarova, Michael Paprota, David Buffa, Peter Kovac, Vit Zmatlo

**Affiliations:** ^1^ Laboratory of Haemato‐oncology and Clinical Biochemistry Hospital Havirov Havirov Czech Republic; ^2^ Department of Pathology Hospital Havirov Havirov Czech Republic; ^3^ Institute of Pathology and Molecular Pathology University Clinic Wuppertal Wuppertal Germany; ^4^ Department of Computer Science Faculty of Electrical Engineering and Computer Science Technical University of Ostrava Ostrava Czech Republic; ^5^ Department of Immunology Faculty of Medicine and Dentistry Palacky University and University Hospital Olomouc Olomouc Czech Republic; ^6^ Department of Haemato‐oncology Faculty of Medicine and Dentistry Palacky University and University Hospital Olomouc Olomouc Czech Republic; ^7^ Department of Clinical Haematology University of Ostrava and University Hospital Ostrava Ostrava Czech Republic; ^8^ Department of Haemato‐oncology University of Ostrava and University Hospital Ostrava Ostrava Czech Republic

1

Dear Editor,

In our research, we showcased that AI‐driven automated digital morphology (ADM), with its innovative categorisation options and ability to visually represent cellular contexts, opens up unprecedented avenues for bone marrow (BM) cellular classification in haemato‐oncology, supporting clinical decisions. However, despite its remarkable image quality and clinical consistency, particularly in reactive haemopoiesis and myeloproliferative and myelodysplastic neoplasms, certain neoplastic BM cells are notably challenging for ADM to classify, which can significantly affect BM cytomorphological diagnosis.

Correct cytomorphological evaluation of BM smears remains a cornerstone of diagnostics in haematology with critical clinical impact.^1^, ^2^ Current expert optical microscopy is limited by substantial subjective interobserver variability and the need for highly skilled cytomorphologists. Furthermore, expert findings may not be considered definitive or the only possible correct results, especially within closely related borderline categories or when dealing with ambiguous cellular classifications. Therefore, innovative objective digital technologies for BM cytomorphology are vitally needed.^1^, ^2^, ^3^, ^4^ There is limited data on the utility of ADM in this field.^3^, ^4^

Our study of a real‐world cohort of 328 BM smears of European ancestry patients aims to comprehensively assess the effectiveness, reliability, and limitations of AI‐assisted ADM (Morphogo system) by comparing it with expert optical microscopy (Figure 1). The cases were divided into six diagnostic groups: myelodysplastic neoplasms (MDN: 15%), multiple myeloma (MM: 14%), mature B/T‐cell neoplasms (B/T‐lymphoma: 13%), acute leukaemia and chronic myelomonocytic leukaemia (AL+CMML: 9%), myeloproliferative neoplasms (MPN: 8%), and reactive haemopoiesis (reactive: 41%). High‐resolution digital images (magnification 1000×) of 500 BM nucleated cells were acquired. ADM's cell recognition (classification) capabilities were evaluated by asking it to correctly classify cells into one of 25 categories, compared with independent consensual expertise with double reading. The percentage of relevant correctly classified cells out of all classified cells was 95.4%. Satisfactory correlation values, as indicated by the Matthews correlation coefficient (MCC), were .400 or above for 22 out of 25 (88.0%) cell types; unsatisfactory MCC values below  .400 were noted for 3 out of 25 (12%) cell types (lymphoblasts, prolymphocytes, and promonocytes) (Figure S4).

**FIGURE 1:**
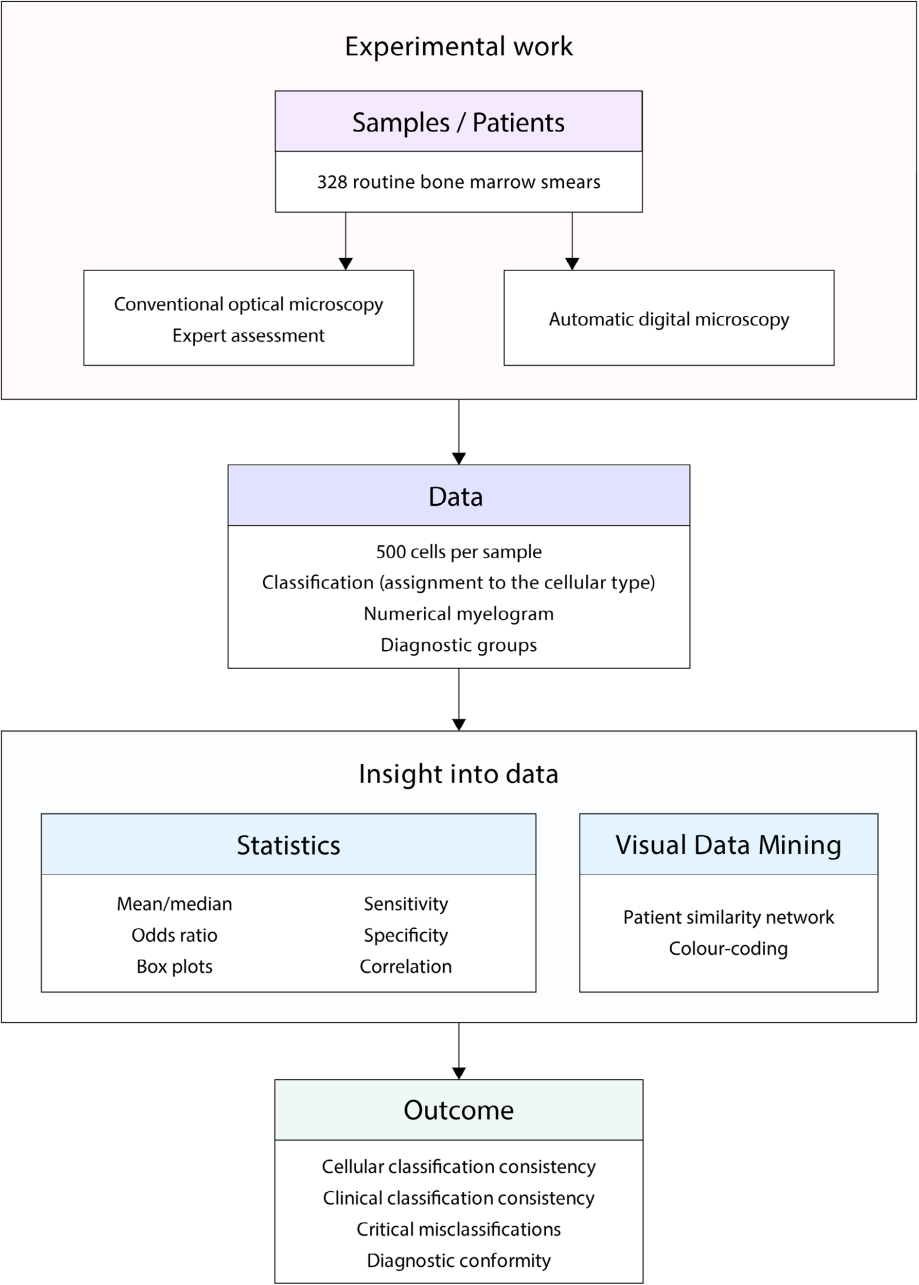
Design of the study. Conventional optical microscopy and automated digital morphology were performed in 328 bone marrow smears. Data for the analysis included image information concerning all analysed cells, results of the classification and numerical myelograms. After dividing patients into six clinically relevant diagnostic groups, standard statistical methods and visual data mining were applied. Cellular and clinical classification consistency were assessed, and critical misclassifications were identified. The data were visualised using the patient similarity network.

Next, we evaluated the percentage of correctly classified cells in individual patients for clinical consistency. The overall relevant clinical consistency was 97.1% (median), with 94.5% of cases showing consistency rates between 80% and 100%. In 5.5% of patients, critical misclassification was noted, with relevant clinical consistency values ranging from 36% to 79%, due to failure to recognise neoplastic cells. Errors included misclassifying neoplastic lymphocytes as blasts; small lymphoblasts/myeloblasts as lymphocytes; granular monoblasts as promyelocytes; and dysplastic monocytic lineage elements, plasmablasts, and immature plasma cells, all exhibiting atypical cytomorphology (Figure 2). Within the B/T‐lymphoma group, misclassified lymphocytes were medium‐sized, featuring abundant basophilic or pale cytoplasm with projections and finer chromatin, sometimes with a nucleolus or presenting a blastoid appearance, unlike correctly classified neoplastic cells. In the AL+CMML group, the misclassified lymphoblasts/myeloblasts were small, displayed a high nucleoplasmic ratio, and had coarser chromatin and nucleoli. Numerous granular monoblasts were misclassified as promyelocytes. In CMML, misclassifications involved the monocytic lineage. In MM, misclassifications were noted in the plasma cell lineage (plasmablasts and immature plasma cells) (Figure 2). The patient similarity network^5^ was constructed to visualise differences between patients and highlight cases with misclassifications (Figure 3). Patients with critical misclassifications with misdiagnosis potential were identified only in three diagnostic categories: AL+CMML, MM, and B/T‐lymphoma. None were found in the MPN, MDN, or reactive groups (Figures 2 and 3). In addition to cytomorphological overlap, misclassifications likely reflected the rarity of some diagnoses and expert inconsistency for certain cells, resulting in incorrect annotations and gaps in training data.

**FIGURE 2:**
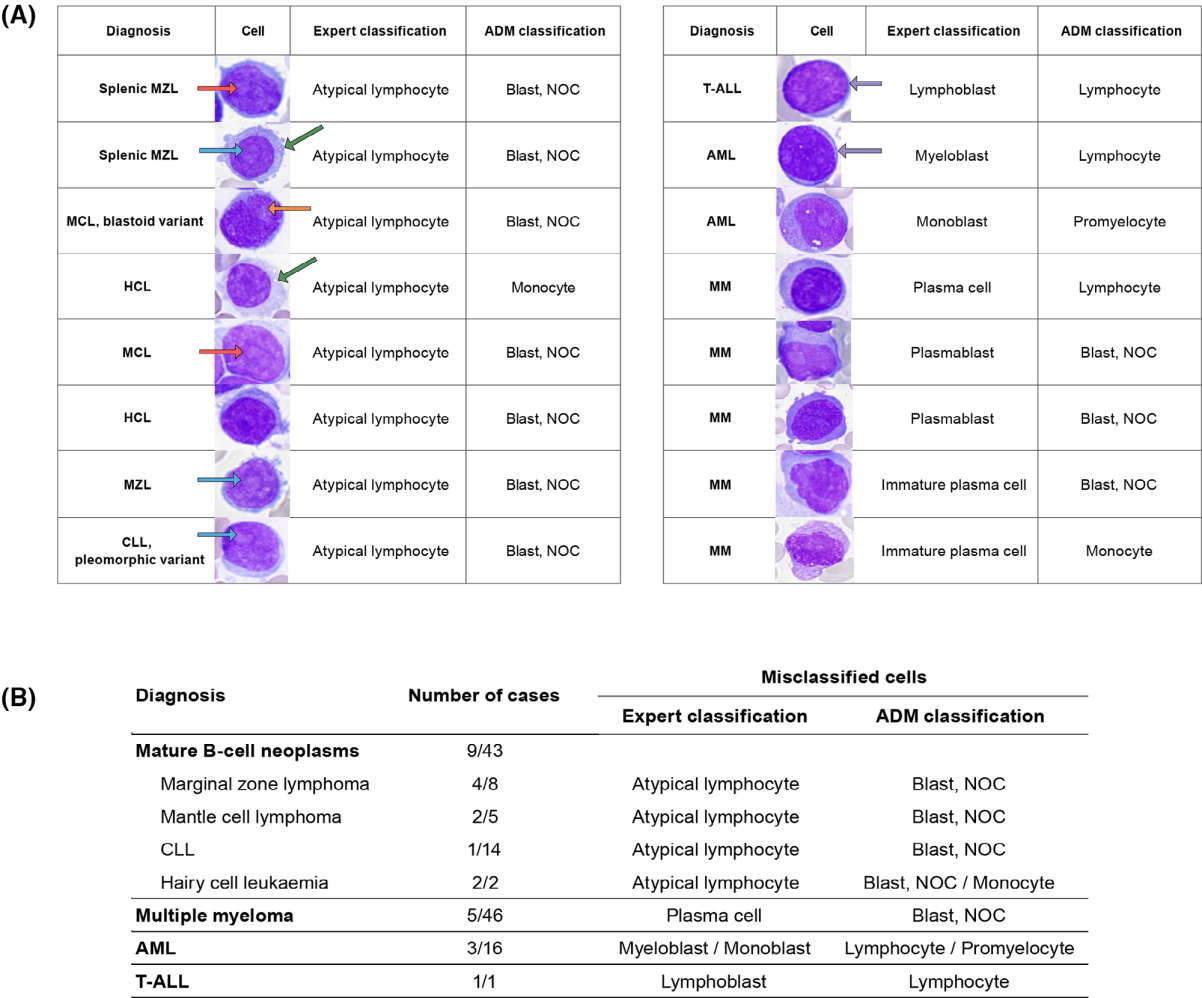
(A) Representative examples of the most frequent misclassifications of BM cells in patients with haematological neoplasms. Misclassified neoplastic lymphocytes, myeloblasts, lymphoblasts, monoblasts and plasma cells are shown in certain cases of marginal zone lymphoma (MZL), mantle cell lymphoma (MCL), hairy cell leukaemia (HCL), chronic lymphocytic leukaemia (CLL), acute myeloid leukaemia (AML), acute T‐lymphoblastic leukaemia (T‐ALL) and multiple myeloma (MM). The confusion of atypical lymphocyte/blast was the most frequent misclassification. The main cytomorphological features of misclassified neoplastic lymphocytes were: medium size, finer chromatin (red arrows), nucleoli (blue arrows), abundant basophilic or pale cytoplasm with projections (green arrows), blastoid appearance (orange arrow). Misclassified lymphoblasts and myeloblasts were small, with a high nucleoplasmacytic ratio and coarser chromatin with nucleoli (grey arrows). Plasmablasts and immature plasma cells were misclassified as lymphocytes or their affiliation to the plasma cell lineage was not recognized (blast, NOC). Cell images were extracted from the MorphogoReview software (version 1.0.4 and 1.0.6). (B) Critical misclassifications by ADM. A critical misclassification was defined as a case in which the individual clinical classification consistency value was below 80%. The clinical group of critical misclassifications by ADM included nine out of 43 patients with mature B‐cell neoplasms, five out of 46 patients with MM, three out of 16 patients with AML and one (out of one) patient with T‐ALL. These patients are also shown in green in Figure 3B. Diagnostically relevant cells, whose incorrect categorisation resulted in critical misclassification, are listed. NOC: not otherwise specified.

**FIGURE 3:**
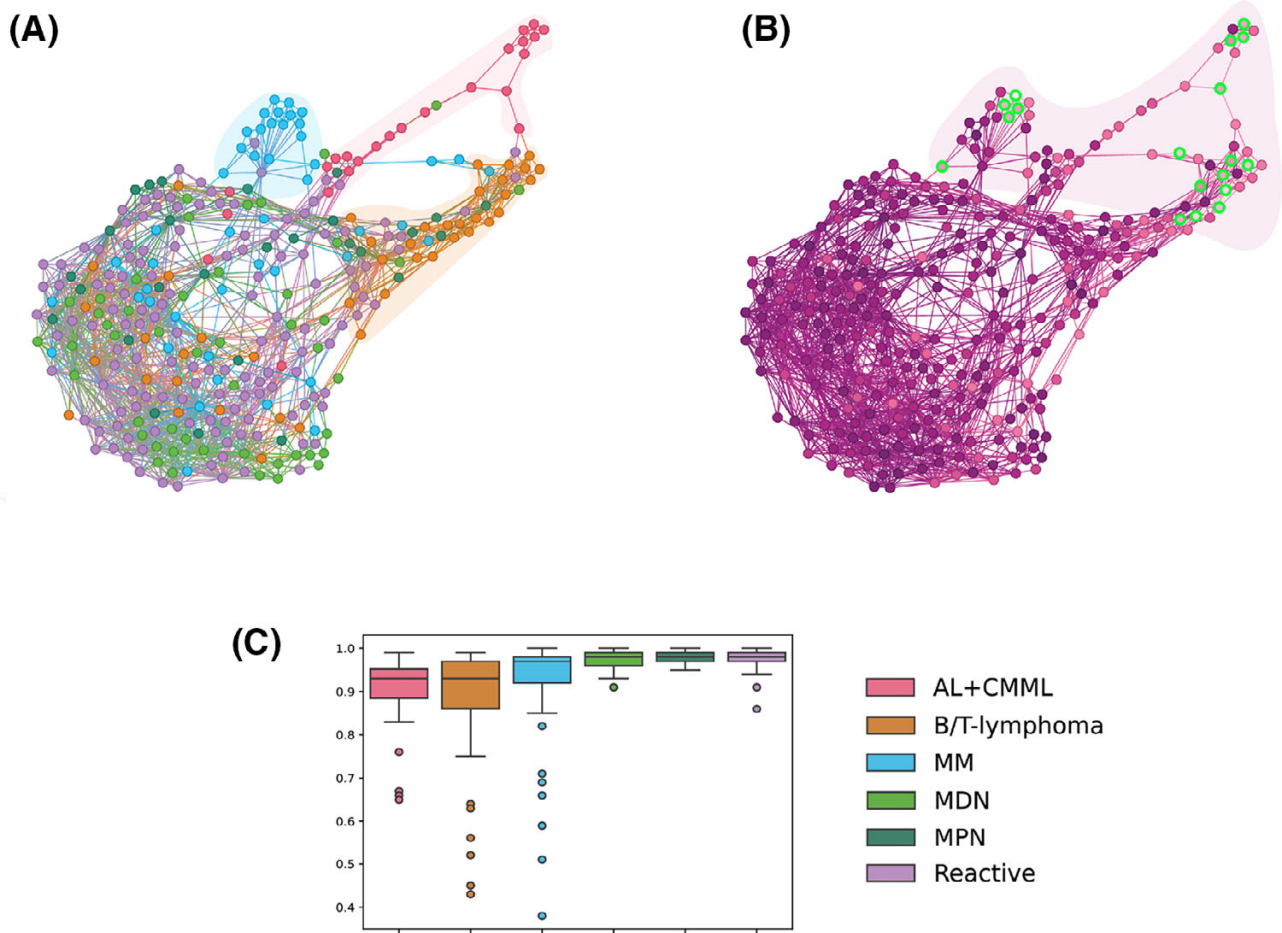
The comparison of numerical differences between optical and digital myelograms and relevant clinical consistency across six diagnostic groups of haematological disorders. (A) Patient similarity network (PSN). First, we calculated numerical differences between expert and ADM evaluation for 25 cell types and selected the clinically relevant cell types. The PSN was constructed based on similarities of patients considering the differences between expert and ADM for the selected cell types. Each dot (network vertex) represents an individual patient, patients closer together share more similar myelogram discrepancies, whether they are lower or higher. The diagnostic groups of all cases are colour‐coded. Three diagnostic subgroups of AL+CMML (red), MM (blue) and B/T‐lymphoma (orange) groups were separated from the other patients in contrast to MDN (light green), MPN (dark green) and reactive (violet) groups. (B) Relevant clinical consistency values in the PSN. In the constructed PSN, the values of relevant clinical consistency are shown in purple. Low values of relevant clinical consistency are shown in light purple, and high values of relevant clinical consistency are shown in dark purple. Patients with critical misclassifications highlighted in green occur exclusively in the diagnostic groups AL+CMML, MM and B/T‐lymphoma. (C) Display of relevant clinical consistency values in the diagnostic groups. The box plot view confirms the visualisation in the form of a network. Patients with low relevant clinical consistency occur predominantly in the diagnostic groups ALL+CMML, MM and B/T‐lymphoma, including all patients with critical misclassifications.

Furthermore, we compared the numerical results of the myelogram from conventional analysis with those obtained from expert ADM, as these are crucial for diagnostics. The most significant numerical differences in the representation of diagnostically relevant cells (blasts, monocytes, lymphocytes, and plasma cells) were observed in AL+CMML for blast counts, AL+CMML for monocyte counts, B/T‐lymphoma for lymphocyte counts, and MM for plasma cell counts (Figure 4). This discovery prompts a thought‐provoking inquiry into the precision of measuring essential diagnostic features and the overall legitimacy of expert assessments. The rigorously standardised procedure for automatically selecting the adaptive area for 1000× immersion‐lens analysis in ADM is a significant factor contributing to numerical discrepancies. ADM can offer greater reliability than traditional light microscopy's subjective and variable area selection.

**FIGURE 4:**
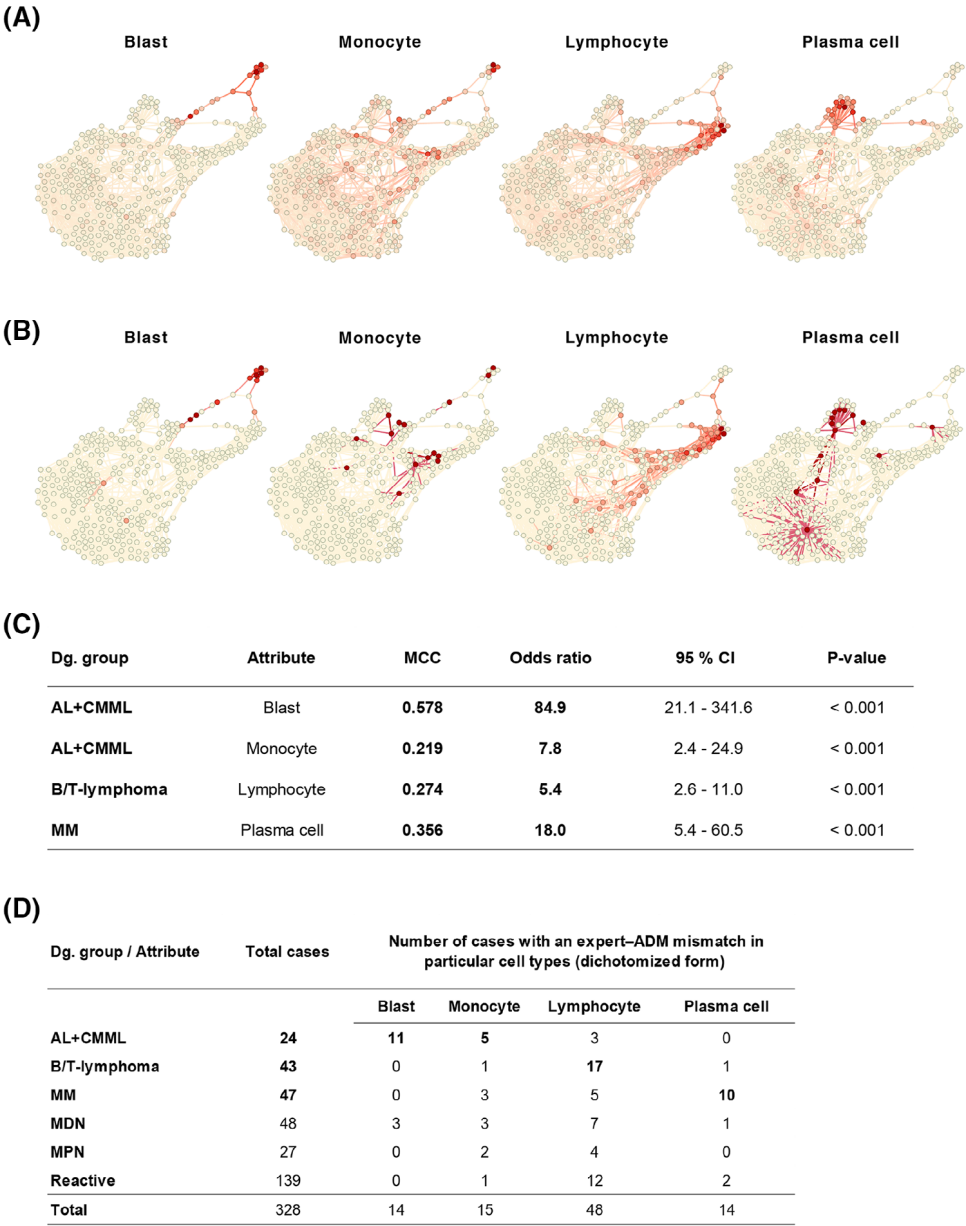
The comparison of numerical differences of key attributes between optical and digital myelograms across six diagnostic groups of haematological disorders. (A, B) The key diagnostically relevant attributes (blast, monocyte, lymphocyte and plasma cell) were selected to show the numerical differences between the optical and ADM myelograms in the patient similarity network (PSN). The largest differences in the representation of blasts occurred in AL+CMML, monocytes in AL+CMML, lymphocytes in B/T‐lymphoma and plasma cells in MM groups. This applies to both the original values (4A) and their scaling (4B). (C) MCC and odds ratio (OR) values were calculated for the key attributes in the diagnostic groups AL+CMML, MM and B/T‐lymphoma, confirming the findings from the PSN; despite the favourable OR, two of them with MCC < .4 lack sufficient classification power. (D) Occurrence of cases with an expert–ADM mismatch. For each of the six diagnostic groups (rows), the prevalence of high differences is shown, with a non‐zero value for the corresponding scale (columns); the occurrences used for further analysis are in bold. These attributes have a non‐negligible relative prevalence for the diagnosis in relation to the total number of cases.

There are differences between the results of our analysis and prior studies. Unlike our cohort with 59% haematolymphoid tumours including rare diagnoses, the rate of neoplasms was under 30% in the Chinese study,^3^ with approximately one‐fifth of the BM smears categorised as ‘relatively normal’. The representation of B/T‐lymphomas in that study was 2.4%, compared with 13% in our cohort. The differences are also attributable to the number of BM samples analysed and the methodology employed to assess classification consistency. Our novel clinical approach, which takes individual patients into account, alongside the use of patient similarity networks to visually evaluate the dataset, is highly relevant to BM cytomorphological diagnosis and yields different results compared with an approach focused solely on cellular classification.

Regarding the perspectives, further, AI training focused on specific cell types is essential to improving classification consistency, enabling the subclassification of lymphomas into specific diagnostic entities,^6^ analysing elements of megakaryopoiesis, detecting metastatic cells in BM,^7^, ^8^, ^9^ and facilitating reliable recognition of lineage dysplasia.^10^

In summary, the data clearly shows that ADM is a highly beneficial diagnostical method for BM cytomorphology. The approach has the potential to revolutionise and improve diagnostics by minimising subjectivity and variability in evaluations. Our research is the first to identify and describe the cell misclassifications in detail, adversely affecting BM cytomorphological diagnosis and undermining the system's reliability. Consequently, this study highlights candidate cells for future AI training and testing. The results of the study also point to a possible (and ongoing) limitation of the use of AI in this case. Any negative consequences can be effectively addressed through expert supervision, underscoring the crucial role of highly trained morphologists in ensuring accurate cell classification and diagnostic interpretation in haemato‐oncology and driving innovation to reach full potential.

## AUTHOR CONTRIBUTIONS

David Starostka: Conceptualisation and design of the study, comprehensive BM diagnostics, expert review of pre‐classification, data management, statistical analysis and initial drafting of the manuscript. Richard Dolezilek: Conceptualisation and design of the study, comprehensive BM diagnostics and initial drafting of the manuscript. Hans Michael Kvasnicka: Conceptualisation and design of the study and review of the manuscript draft. Milos Kudelka: Contribution to the design of the study, statistical analysis and review of the manuscript draft. Petra Miczkova: Preparation and scanning of BM slides, expert review of pre‐classification, data management and review of the manuscript draft. Eva Kriegova: Contribution to the design of the study and review of the manuscript draft. David Kolacek: Comprehensive BM diagnostics, data management and review of the manuscript draft. Barbora Sotkovska and Jarmila Juranova: Preparation and scanning of BM slides, data management and review of the manuscript draft. Tomas Anlauf: Statistical analysis and review of the manuscript draft. Katerina Chasakova, Michael Paprota and Peter Kovac: Data management and review of the manuscript draft. Sona Kolarova: Review of the manuscript draft. David Buffa: Data management and review of the manuscript draft. Vit Zmatlo: Graphics and review of the manuscript draft. All authors have made a significant contribution to this study and have approved the final manuscript.

## CONFLICT OF INTEREST STATEMENT

The authors declare no conflict of interest.

## FUNDING INFORMATION

The study was supported by the Internal Research Grant 2023 of Hospital Havirov, and in part by the Ministry of Health of the Czech Republic (FNOl 0098892).

## 3 ETHICS STATEMENT

The study was approved by the Local Ethics Committee of the Hospital Havirov and carried out in accordance with the updated principles of the Helsinki Declaration. The study used archived material. The patients gave their written informed consent for BM examination and anonymous data collection and analysis.

## Supporting information



Supporting Information

## Data Availability

The data that support the findings of this study are available from the corresponding author upon reasonable request.
