# Supplementary material for: The utility of automated artificial intelligence‐assisted digital cytomorphology for bone marrow analysis in diagnostic haemato‐oncology
Source: Clin Transl Med. 2025 Jul 9;15(7):e70364. doi: 10.1002/ctm2.70364 (PMC12238674; doi:10.1002/ctm2.70364)
Supplement: Supplementary file 1 — Supporting Information [file CTM2-15-e70364-s001.docx]

**The utility of automated artificial intelligence–assisted digital cytomorphology for bone marrow analysis in diagnostic haemato-oncology**

**Automated digital morphology of bone marrow**

David Starostka^1^, Richard Dolezilek^2^, Hans Michael Kvasnicka^3^, Milos Kudelka^4^, Petra Miczkova^1^, Eva Kriegova^5^, David Kolacek^1^, Barbora Sotkovska^1^, Tomas Anlauf^4^, Jarmila Juranova^6^, Katerina Chasakova^1^, Sona Kolarova^7^, Michael Paprota^1^, David Buffa^8^, Peter Kovac^2^, and Vit Zmatlo^1^

^1^Laboratory of Haemato-oncology and Clinical Biochemistry, Hospital Havirov, Havirov, Czech Republic; ^2^Department of Pathology, Hospital Havirov, Havirov, Czech Republic; ^3^Institute of Pathology and Molecular Pathology, University Clinic Wuppertal, Wuppertal, Germany; ^4^Department of Computer Science, Faculty of Electrical Engineering and Computer Science, Technical University of Ostrava, Ostrava, Czech Republic; ^5^Department of Immunology, Faculty of Medicine and Dentistry, Palacky University and University Hospital Olomouc, Olomouc, Czech Republic; ^6^Department of Haemato-oncology, Faculty of Medicine and Dentistry, Palacky University and University Hospital Olomouc, Olomouc, Czech Republic; ^7^Department of Clinical Haematology, University of Ostrava and University Hospital Ostrava, Ostrava, Czech Republic; ^8^Department of Haemato-oncology, University of Ostrava and University Hospital Ostrava, Ostrava, Czech Republic

**Correspondence**

David Starostka, Laboratory of Haematooncology and Clinical Biochemistry, Hospital Havířov, Dělnická 1132, 73601 Havířov, Czech Republic. Tel: +420-777 117 886, Fax: +420-596 491 536, E-mail: [david.starostka@nemhav.cz](mailto:david.starostka@nemhav.cz) ORCID: <https://orcid.org/0000-0002-3113-8032>

***Declarations***

**Authorship contributions**

D.S. – conceptualisation and design of the study, comprehensive BM diagnostics, expert review of pre-classification, data management, statistical analysis, initial drafting of the manuscript; R.D. – conceptualisation and design of the study, comprehensive BM diagnostics, initial drafting of the manuscript; H.M.K. – conceptualisation and design of the study, review of the manuscript draft; M.K. – contribution to the design of the study, statistical analysis, review of the manuscript draft; P.M. – preparation and scanning of BM slides, expert review of pre-classification, data management, review of the manuscript draft; E.K. – contribution to the design of the study and review of the manuscript draft; D.K. – comprehensive BM diagnostics, data management, review of the manuscript draft; B.S. – preparation and scanning of BM slides, data management, review of the manuscript draft; T.A. – statistical analysis, review of the manuscript draft; J.J. – preparation and scanning of BM slides, data management, review of the manuscript draft; K.CH. – data management, review of the manuscript draft; S.K. – review of the manuscript draft; M.P. – data management, review of the manuscript draft; D.B. – data management, review of the manuscript draft; P.K. – data management, review of the manuscript draft; V.Z. – graphics, review of the manuscript draft.

All authors have made a significant contribution to this study and have approved the final manuscript.

**Disclosure of Conflicts of Interest**

Authors have no conflict of interests related to this publication.

**Acknowledgments**

The study was supported by the Internal Research Grant 2023 of Hospital Havirov, and in part by the Ministry of Health of the Czech Republic (FNOl 0098892).

**Ethical approval**

The study was approved by the Local Ethics Committee of the Hospital Havirov and carried out in accordance with the updated principles of the Helsinki Declaration. The study used archived material. The patients gave their written informed consent for BM examination and anonymous data collection and analysis.

**Data Sharing Statement**

The data that support the findings of this study are available from the corresponding author upon reasonable request.

***Abstract***

The clinical utility of automated digital bone marrow (BM) cytomorphology has not been fully explored. Therefore, our study aimed to comprehensively assess the effectiveness, reliability and limitations of this innovative method. In diagnostic BM smears from 328 patients with haematological disorders, we compared BM cell quantification and recognition using an automated digital morphology system (ADM; Morphogo) assisted by artificial intelligence with expert optical microscopy. Besides high image quality, the overall correct cell classification was 95.4%; only three out of 25 cell types were systematically misclassified. Clinical consistency reached 97.1%, with 98.0% in reactive haemopoiesis, myeloproliferative and myelodysplastic neoplasms. In acute leukaemia/chronic myelomonocytic leukaemia (AL/CMML), B/T-lymphomas and multiple myeloma (MM) groups, clinical consistency below 80% occurred in 18 out of 328 (5.5%) patients due to critical misclassification of atypical neoplastic lymphocytes, blasts, promonocytes/monocytes and immature plasma cells. Listed BM neoplastic cells remain difficult to classify, and advancements are needed for reliable diagnostics. Comparing ADM and optical microscopy, the largest numerical differences in the representation of blasts were observed in AL/CMML, monocytes in AL/CMML, lymphocytes in B/T-lymphoma, and plasma cells in MM groups. It raises a question regarding the accuracy of quantifying key diagnostic cells. The method used in this study has enormous potential to drive diagnostic transformation by reducing subjectivity and variability in assessments.

***Key words***

Automated digital morphology, artificial intelligence, bone marrow, diagnosis, haemato-oncology

1. ***Introduction***

The correct cytomorphological evaluation of peripheral blood (PB) and bone marrow (BM) smears remain a cornerstone of multidisciplinary diagnostics in haemato-oncology, influencing critical clinical decision points.^1–3^ Optical microscopy is the gold standard method in the cytomorphological analysis of blood cells, requiring high magnification imaging (100× oil immersion lenses).^1, 2, 4^ This labour-intensive and time-consuming analysis requires the expertise of highly skilled, experienced professionals, and it is accompanied by natural intra-expert and inter-expert variability related to the subjectivity of the evaluation. Therefore, innovative supporting digital technologies are vitally needed.^1, 4–6^

Unprecedented developments have been made in automated image classification and decision making supported by artificial intelligence (AI). In haematology, automated digital morphology (ADM) is currently used mainly for PB smears for cell classification, primary diagnosis and second opinion.^2–4^ Integrating advanced whole slide imaging with cell recognition and classification algorithms based on convolutional neural networks (CNNs), represents a natural evolution also in cytomorphological BM analysis.^7–10^ Compared with PB, ADM presents challenges in BM due to its complex and intricate cytomorphological pattern, the high number of different cell types and the similarity of precursors. However, the diagnostic impact of BM analysis remains crucial in haematological malignancies.

Currently, AI in ADM facilitates novel ways of interpreting image data within the context of the cellular populations analysed.^1, 7–9^ Despite its perspectives in terms of diagnostic efficiency, ADM of BM is not currently routinely used, due to limited training datasets, lack of validation and systematic errors and bias.^10–14^

Our study aims to assess the effectiveness and reliability of an ADM system (Morphogo) using a retrospective real-world cohort of 328 BM smear samples, comparing it with a conventional optical method and conducting expert assessment. In patients with various haematopoietic disorders, we focused on evaluating the system’s cellular and clinical classification consistency. We compared conventional optical expert-based approach with digital myelograms. We studied the limitations in cell classification and diagnostic conformity, as well as the potential for improving analytical and diagnostic outcomes for clinical practice in diagnostic haemato-oncology. Compared with prior works, in our real-world cohort with haematological malignancies and reactive BM, we applied a novel methodology that emphasised clinical relevance and used visual interpretation of data with the aid of patient similarity networks (PSNs). This approach yielded more clinically interpretable results and enabled accurate identification of diagnostically substantial misclassified cells.

***2. Mate rials and methods***

**2.1 Study design**

This study compared the results of ADM with expert optical microscopy based on a 500-cell count for each sample, following the International Council for Standardisation in Haematology’s (ICSH’s) recommendations.^15^ The study design is depicted in Figure S1.


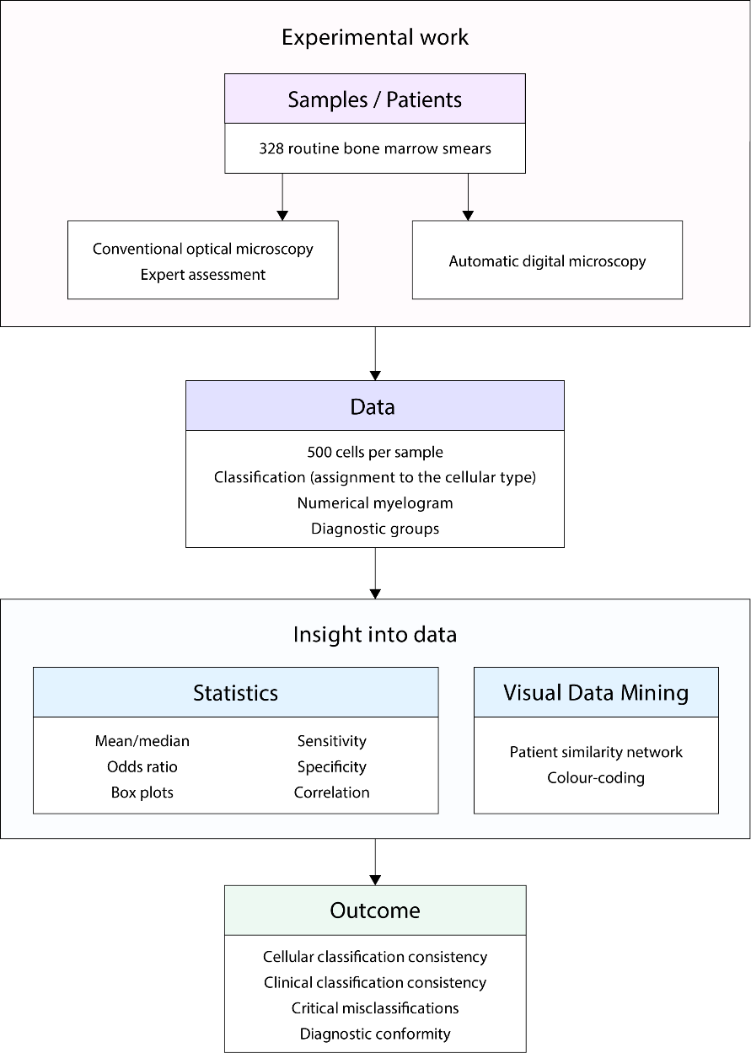


***Figure S1. Design of the study.*** Conventional optical microscopy and automated digital morphology were performed in 328 bone marrow smears. Data for the analysis included image information concerning all analysed cells, results of the classification and numerical myelograms. After dividing patients into six clinically relevant diagnostic groups, standard statistical methods and visual data mining were applied. Cellular and clinical classification consistency and diagnostic conformity were assessed, and critical misclassifications were identified.

**2.2 Cohort characteristics**

The retrospective real-world Caucasian cohort included 328 routine Giemsa-stained BM smears from two diagnostic laboratories taken at diagnosis from January 2022 to September 2023. In the cohort (54% males and 46% females), patients were aged 30–90 years (median = 71 years). In all patients, one BM smear has been assessed. None of the selected samples were excluded due to poor-quality BM smear scans.

**2.3 Conventional cytomorphological BM analysis**

The conventional myelogram was determined by qualified and highly experienced laboratory technicians via optical microscopy using high-quality Nikon Eclipse and Olympus BX53 instruments equipped with 100× immersion lenses. Staining and a 500-cell count were applied for each sample, following ICSH recommendations.^15^ Only Giemsa-stained smears were included, as this staining is standard for BM cytomorphology throughout Europe, in accordance with ICSH guidance.^15^ The final diagnosis, based on the current version of the WHO classification¹⁶, was confirmed by a multidisciplinary board. An identical smear was used for optical microscopy and ADM.

**2.4 ADM: Morphogo system workflow**

The CNN-based Morphogo system (Hangzhou ZhiWei Information Technology Co., Ltd, Hangzhou, China) was employed in the study to conduct BM ADM. The device can fully scan PB and BM smears and then locate, precisely record and pre-classify nucleated cells and release a myelogram.

All BM smears were scanned at high magnification (1000×) using an oil lens. A 500-cell count was applied for each sample, following ICSH recommendations.^15^ The cell recognition and assignment (designated as classification^13, 17–19^) was set to 25 types: proerythroblast, early erythroblast (including megaloblastic), intermediate erythroblast (including megaloblastic), late erythroblast (including megaloblastic), myeloblast, promyelocyte, neutrophilic myelocyte, neutrophilic metamyelocyte, band neutrophil, segmented neutrophil, eosinophilic myelocyte, eosinophilic metamyelocyte, band eosinophil, segmented eosinophil, basophil, monoblast, promonocyte, monocyte, lymphoblast, prolymphocyte, mature lymphocyte (including atypical and reactive), plasmablast, immature plasma cell, plasma cell and others (smudge cell, histocyte, phagocyte, mast cell and mitosis).

The Acquisition Terminal managed the scanning process, ensuring efficient image acquisition, while the Review Terminal facilitated the review process. Two qualified and highly experienced experts in cytomorphology then independently reviewed and adjusted the pre-classification results locally or remotely using the MorphogoReview software (version 1.0.4 and 1.0.6). Each case was reviewed by both experts, with matching classification in >99% of cells. For cells with differing classifications (<1%), inter-observer consensus was reached.

**2.5 The classification consistency of the ADM system and its critical limitations**

The principle of the consistency analysis was a comparison of the automatic pre-classification of each cell and the final expert classification of each cell; the consensual expert classification was considered as the true classification reference (ground truth).

***2.5.1 Relevant and irrelevant cell misclassifications***

Both cellular and clinical (case) consistency calculations considered relevant and irrelevant cell misclassifications (Figure S2). Irrelevant misclassifications are diagnostically neutral and considered acceptable, i.e. they do not affect the correct diagnosis. These include reciprocal misclassification of lymphoblast/myeloblast/monoblast; neutrophilic, eosinophilic and basophilic promyelocyte/myelocyte; myelocyte/metamyelocyte; metamyelocyte/band; band/segment; proerythroblast/early erythroblast; early/intermediate and intermediate/late erythroblast; promonocyte/monocyte; prolymphocyte/lymphocyte; plasmablast/immature plasma cell; and immature plasma cell/mature plasma cell. Similar ‘tolerance classes’ were used in the most recent published study on Morphogo system consistency.¹⁸ All other misclassifications are considered relevant, as they are unacceptable and may have serious diagnostic and clinical consequences.


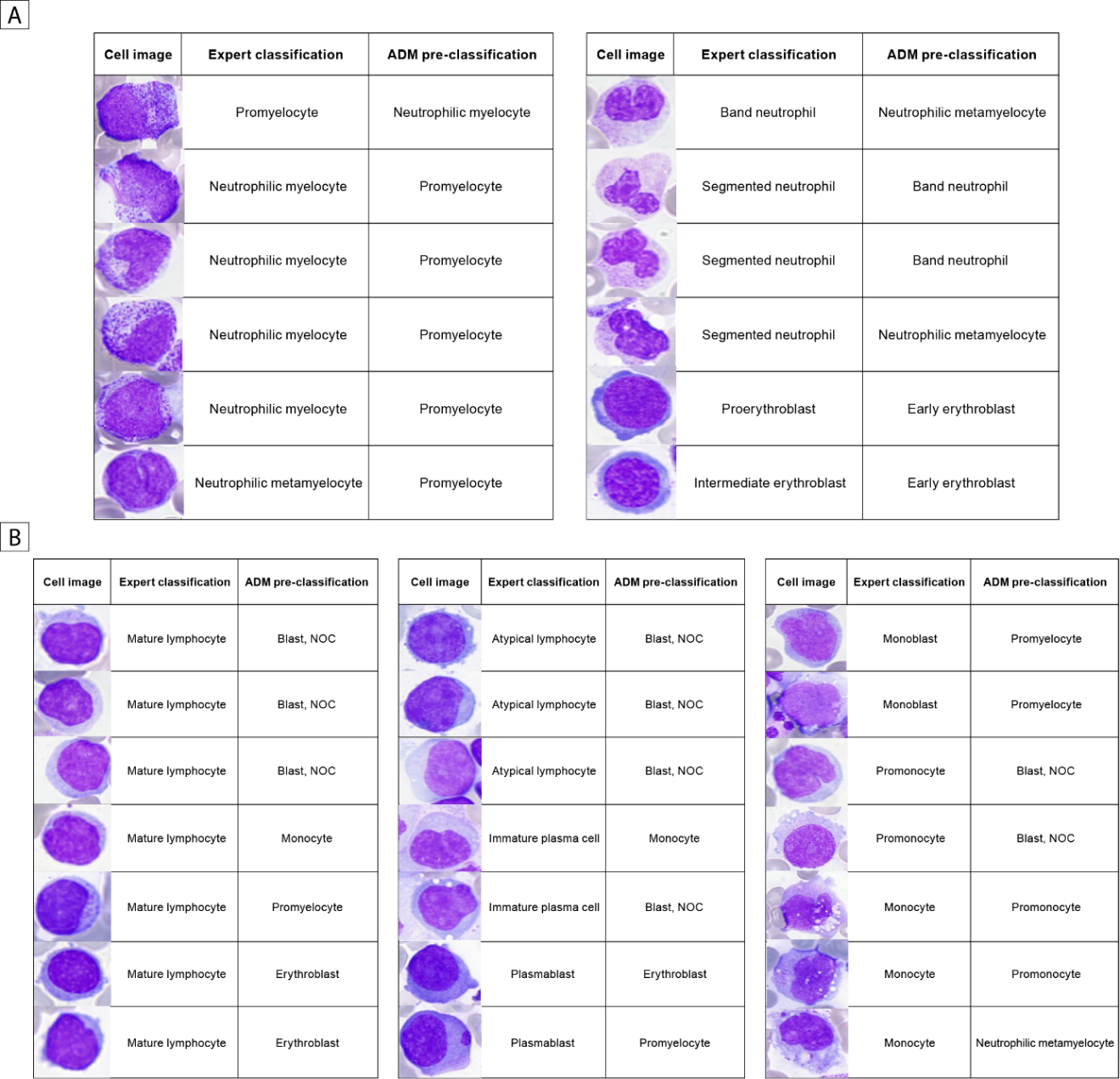


***Figure S2. Irrelevant and relevant cell misclassifications.*** (A) Examples of irrelevant misclassifications in myeloid and erythroid lineages are shown in a comparison between expert classification and Morphogo pre-classification. Cell images were extracted from the MorphogoReview software (version 1.0.4 and 1.0.6). (B) Examples of relevant misclassifications in lymphoid, plasma cell and monocytic lineages are shown in a comparison between expert classification and Morphogo pre-classification. The reference expert classification was facilitated by displaying the cellular context and the proposal of an alternative classification of each cell with the five most likely classification options (‘Top five list’), including the percentage probability, facilitating expert decision-making. The categorisation of some elements was very difficult even for an expert. Cell images were extracted from the MorphogoReview software (version 1.0.4 and 1.0.6).

***2.5.2 The cellular and clinical classification consistency***

The classification consistency was determined using the MorphogoReview software, which evaluated the classification concordance cell by cell. Unclassifiable cells (mainly damaged cells), which were less than 2% of all cells, were eliminated from further analysis. No case with a high incidence of unclassifiable cells was recorded. The percentage of correctly classified cells out of all classified cells was calculated, considering only relevant misclassifications.

The cellular classification consistency was determined from the confusion matrix, which was based on classification consistency for individual cell types. Sensitivity and specificity were calculated for each cell type. Matthews correlation coefficient (MCC) was calculated for each cell type and as an average value and standardly interpreted. An MCC value of 0.400 and above was considered satisfactory.^20^ Cell types with unsatisfactory classification consistency were identified according to the value of MCC.

For each patient, clinical classification consistency was determined as a percentage of correctly pre-classified elements out of all cells evaluated in the case after the elimination of unclassifiable cells. The expert classification was considered the true classification reference. The overall clinical classification consistency was calculated as the median of the individual case values, considering only relevant cell misclassifications.

A critical misclassification was defined as a case in which the individual clinical classification consistency value was below 80%. This arbitrary limit was set by consensus on an expert basis, considering minimal requirements of external quality assessment in cytomorphology rounds.

**2.6 The patient similarity network (PSN)**

The PSN is an innovative approach in biomedicine that enables the comprehensive analysis of biomedical data.^21, 22^ PSNs provide models in which each patient is represented as a vertex, and the links among vertices reflect the similarity of patients based on their characteristics (groups of attributes) under investigation. With simple visualisation, PSNs provide understandable and interpretable results. In visualised PSNs with different densities of connected structures, subgroups of patients with similar characteristics can be identified, and their occurrence in these subgroups can be predicted. Our PSN was constructed by LRNet algorithm^23^ from the ten attributes described below, five of which were scaled (for later visualisation); cosine similarity was used as the similarity measure. To meet specific analytical requirements, the resulting PSN was then visualised in different ways.

For each case, both expert and ADM numerical myelograms were available. First, the difference between expert and ADM assessment (an expert–ADM mismatch*)* was quantified for all cell types and expressed as their absolute values. For further analysis, 10 out of 25 attributes (myeloblast, promyelocyte, monoblast, monocyte, lymphocyte, plasma cell, proerythroblast, early erythroblast, intermediate erythroblast and late erythroblast) with an experimentally confirmed positive influence on the interpretability of structures in the constructed PSN were selected.

Figure S3 shows a visualisation of part of the network constructed from the ten selected attributes. Dots (vertices) represent patients, and lines (edges) represent similarities between pairs of patients. If edges connect several patients in the network and form a cluster together, then these patients are more similar in their attribute values than patients outside this cluster. Visualisation allows patients and their similarities to be highlighted (colour-coded), providing analytically useful information.


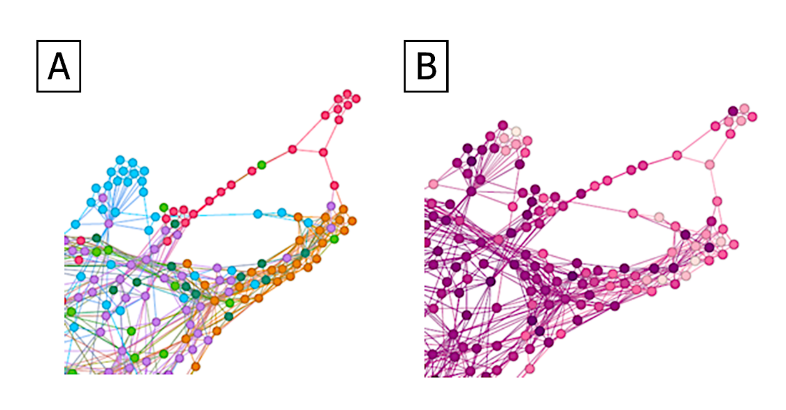


***Figure S3. Colour-coded network fragments with different analytical meanings.*** The visualised network fragments provide different information. In the left fragment (S3A), patients are coloured according to their diagnosis, and in the right part of this fragment, there are three groups of patients with predominantly the same diagnosis (blue, red and orange dots). In the right fragment (S3B), patients are coloured according to the value of the relevant clinical consistency, with higher values corresponding to darker colours. In the right part of the fragment, patients have a lighter colour, indicating a lower value of the relevant clinical consistency.

In the second step, the difference in the higher value was expressed as a percentage relative to the lower value, irrespective of whether the lower value was that of an expert or ADM. The percentage differences in the values of the ten attributes prepared in this way were used to construct the network.

Next, the obtained percentages of selected attributes were linearly scaled into two to five intervals based on thresholds recommended by the domain expert, following the current version of the WHO classification.^16^ To gain deeper insights into the data, we defined the clinically relevant thresholds for each of cell types (myeloblast/monoblast, 3%, 5%, 10% and 20%; monocyte, 10%; lymphocyte, 20%, 40% and 60%; plasma cell, 10%). Based on these thresholds, the results were then scored 0-4 for myeloblasts/monoblasts, 0-1 for monocytes, 0-3 for lymphocytes and 0-1 for plasma cells. In the final scaling, the difference between these scores was determined for each attribute.

In the third step, the scaled attributes were dichotomised so that the values of 0 remained the same and the other values were transformed into 1. The original and rescaled values were then coloured in the visualised PSN, and the dichotomised values were used to calculate the odds ratio and MCC.

**2.7 Odds ratio and Matthews correlation coefficient**

# **Odds ratio (OR)**

The OR is an indicator that quantifies the relationship between two independent binary variables; it is the ratio of the odds of an event occurring in one group to the odds of its occurring in the other group. Even if the OR reaches a high value, it can be misinterpreted, so its confidence interval and *P* value must be considered.

# **Matthews correlation coefficient**

The MCC is a metric used to evaluate the quality of binary classifications not much affected by the problem of unbalanced datasets; it can be understood as a contingency (two-class confusion) matrix method for calculating the Pearson product–moment correlation.^20^ The MCC is in the interval [−1, 1], and although the interpretation can be domain-dependent, a correlation of at least 0.700 is typically considered strong, 0.400 satisfactory (moderate) and 0.200 weak.^20^ Despite reports of the limitations of MCC when used in some unbalanced datasets, MCC still outperforms other measures derived from the confusion matrix.

The Matthews correlation coefficient indicates how well the model can distinguish between the selected diagnosis and other diagnoses in our case. If the value is zero, they cannot be distinguished from each other. The closer the MCC value is to +1, the better the model can distinguish between the selected diagnosis and other diagnoses.

OR and MCC calculations, including 95% CI and P value, were performed in the OMNI calculator (https://www.omnicalculator.com/).

**2.8 Diagnostic conformity of the numerical myelogram**

Diagnostic conformity (concordance) was assessed patient by patient on an expert basis. The binary score expressed the conformity of the conventional numerical myelogram and expert numerical ADM myelogram with the final diagnosis (1 = concordant, 0 = discordant). Overall diagnostic conformity was calculated as the ratio of concordant cases to all evaluated cases as a percentage, both for the optical and the ADM numerical myelogram.

1. ***Results***

**3.1 Cohort characteristics**

In the study cohort composed of 328 patients, the cases were divided into six diagnostic groups: myelodysplastic neoplasms (MDN; 49/328, 15%, including MDN with low blasts and MDN with increased blasts), multiple myeloma (MM; 46/328, 14%, including plasma cell leukaemia), mature B/T-cell neoplasms (B/T-lymphoma; 43/328, 13%, including chronic lymphocytic leukaemia (CLL), follicular lymphoma, mantle cell lymphoma (MCL), marginal zone lymphoma (MZL), hairy cell leukaemia (HCL), lymphoplasmacytic lymphoma and Waldenström macroglobulinaemia, diffuse large B-cell lymphoma and Sézary syndrome), acute leukaemia and chronic myelomonocytic leukaemia (AL+CMML; 30/328, 9%, including acute myeloid leukaemia (AML), acute B-lymphoblastic leukaemia, acute T-lymphoblastic leukaemia and CMML), myeloproliferative neoplasms (MPN; 26/328, 8%, including essential thrombocytaemia, polycythaemia vera, primary myelofibrosis and systemic mastocytosis) and reactive haemopoiesis and monoclonal gammopathy of undetermined significance (reactive, 134/328, 41%).

**3.2 The cellular and clinical classification consistency**

Using ADM, high-resolution digital images (magnification 1000×, 2048 × 1536 pixels) of BM nucleated cells, including all megakaryocytes, were acquired in all BM smears. First, the cell recognition ability of ADM was assessed to assign cells to the correct types out of 25 types (designated as classification). In Figure S4, the cellular classification consistency of ADM is summarised in the confusion matrix, along with the results of the MCC. The percentage of relevant correctly classified cells out of all classified cells was 95.4%. Satisfactory MCC values above 0.400 were observed in 22 out of 25 (88.0%) cell types, whereas very strong MCC values above 0.700 were observed in 18 out of 25 (72.0%) cell types, including 10 out of 25 (40.0%) that were above 0.900. Unsatisfactory MCC values under 0.400 were observed in three out of 25 (12%) cell types (lymphoblasts, prolymphocytes and promonocytes).


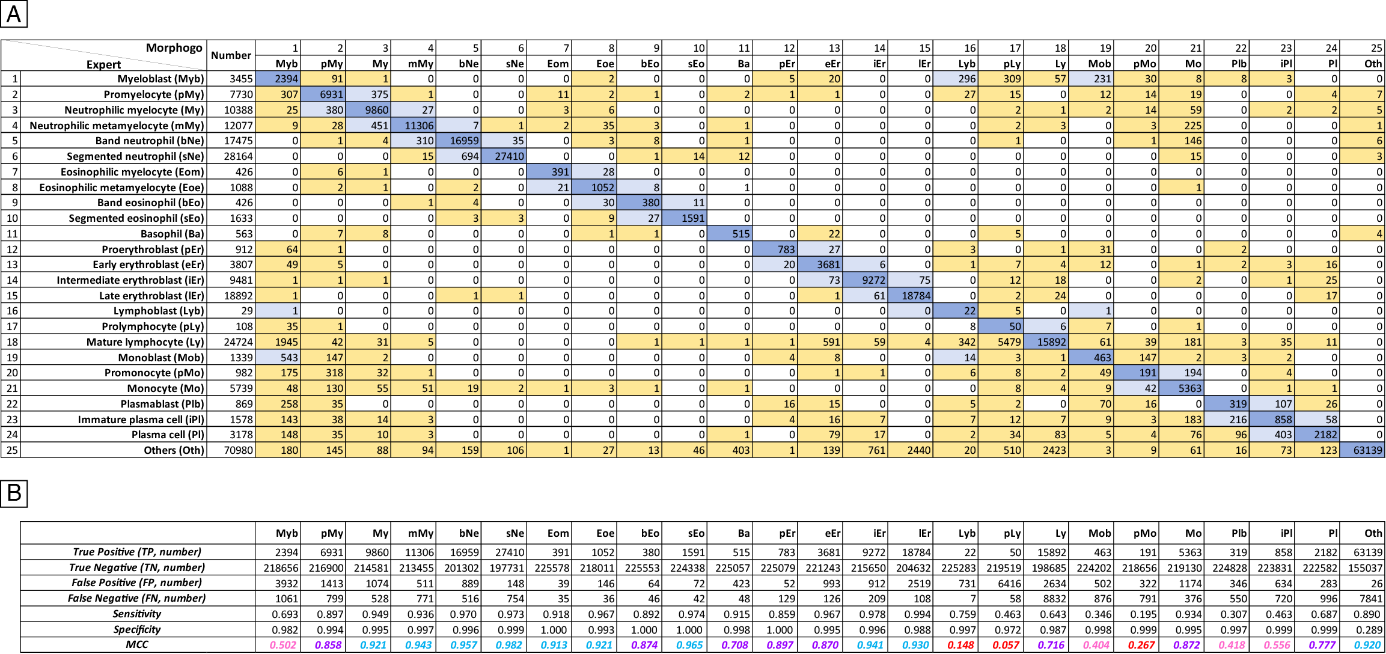


***Figure S4. Cellular classification consistency between optical and automated digital cytomorphology in the bone marrow smears of 328 patients with haematological disorders.*** (A) In the confusion matrix, the rows display the cell classification results of ADM, and the columns show the results of the expert classification. The number of cells is listed in boxes. The dark blue boxes on the diagonal line represent cells classified consistently by ADM and the expert. The light blue boxes represent irrelevant (tolerable) misclassifications. The yellow boxes represent cells misclassified by ADM (relevant misclassifications). (B) The dataset consisted of 500 evaluated nucleated cells per patient after the exclusion of smudge cells. For all cell types, sensitivity, specificity and MCC values are shown. Strong correlation MCC values above 0.700 are marked in violet and blue (excellent, >0.900), satisfactory MCC values (0.400–0.700) are marked in pink and unsatisfactory MCC values below 0.400 are marked in red.

Regarding clinical consistency, the percentage of correctly classified cells in individual patients was assessed, considering relevant and irrelevant misclassifications. Relevant clinical consistency was 97.1% (median), and in 310 out of 328 (94.5%) cases, relevant clinical consistency was 80%–100%.

**3.3 The critical limitations of the classification ability of the ADM system**

In 18 out of 328 (5.5%) patients, critical misclassification with individual values of relevant clinical consistency below 80% (36%–79%) was identified with a recognition failure of neoplastic cells. The correct differentiation of atypical lymphocyte/blast, myeloblast/lymphocyte, lymphoblast/lymphocyte, monoblast+promonocyte/promyelocyte and immature plasma cell/blast were the most frequent errors, all occurring without any flags for expert review in the Morphogo software (Table S1; Figure S5).

***
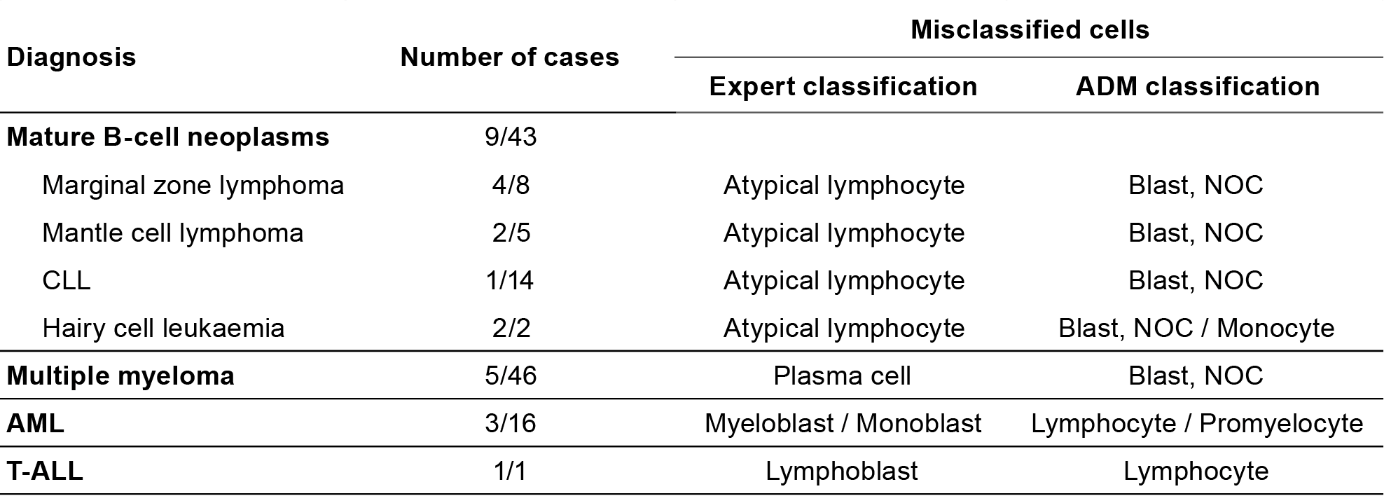
***

***Table S1. Critical misclassifications by ADM.*** A critical misclassification was defined as a case in which the individual clinical classification consistency value was below 80%. The clinical group of critical misclassifications by ADM included nine out of 43 patients with mature B-cell neoplasms, five out of 46 patients with MM, three out of 16 patients with AML and one (out of one) patient with T-ALL. These patients are shown in green in Figure S6B and red, blue and orange dots in Figure S6C. Diagnostically relevant cells, whose incorrect classification resulted in critical misclassification, are listed. NOC: not otherwise specified; CLL: chronic lymphocytic leukaemia.


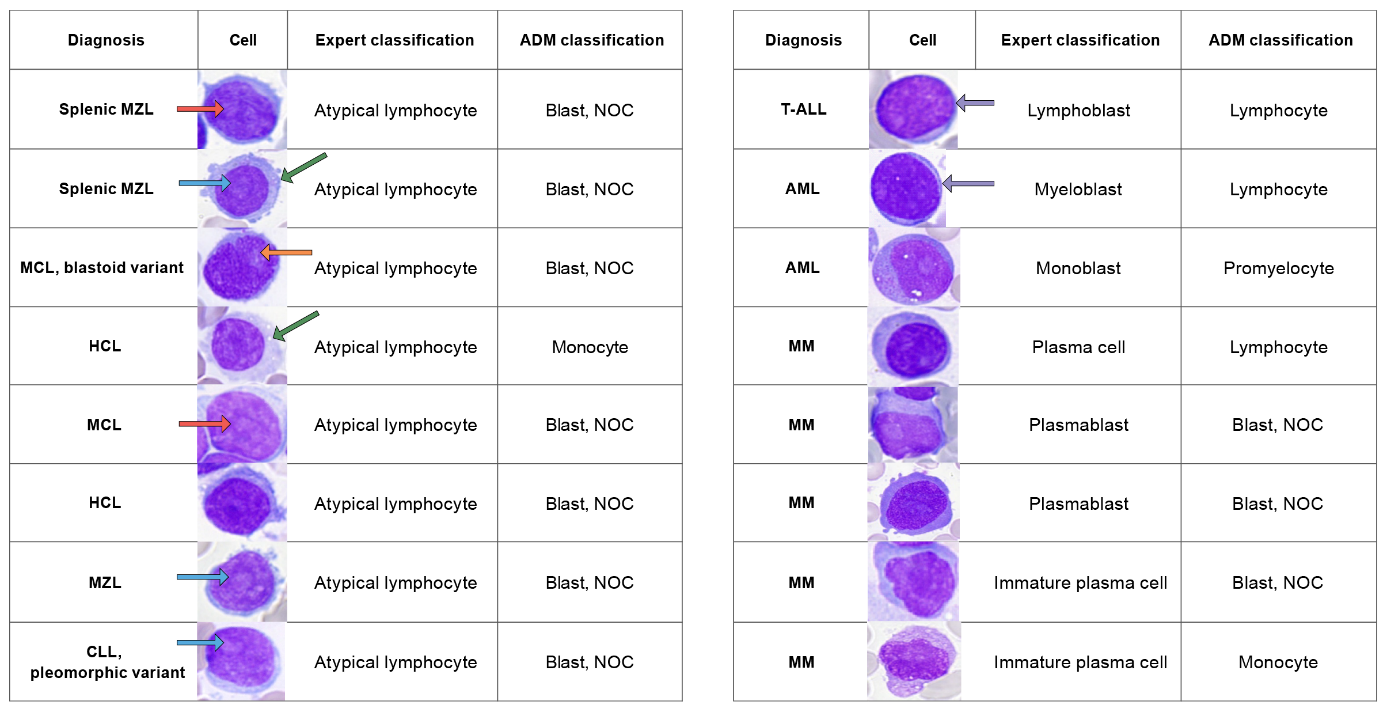


***Figure S5. Representative examples of the most frequent misclassifications of BM cells in patients with haematological neoplasms.*** Misclassified neoplastic lymphocytes, myeloblasts, lymphoblasts, monoblasts and plasma cells are shown in certain cases of MZL, MCL, HCL, CLL, acute myeloid leukaemia (AML), acute T-lymphoblastic leukaemia (T-ALL) and MM. The misclassification of atypical lymphocyte/blast was the most frequent error. The main cytomorphological features of misclassified neoplastic lymphocytes were: medium size, finer chromatin (red arrows), nucleoli (blue arrows), abundant basophilic or pale cytoplasm with projections (green arrows), blastoid appearance (orange arrow). Misclassified lymphoblasts and myeloblasts were small, with a high nucleoplasmacytic ratio and coarser chromatin with nucleoli (grey arrows). Plasmablasts and immature plasma cells were misclassified as lymphocytes or their affiliation to the plasma cell lineage was not recognized (blast, NOC). Cell images were extracted from the MorphogoReview software (version 1.0.4 and 1.0.6).

In patients with critical misclassifications, the morphology of correctly classified and misclassified cells was evaluated. Cytomorphology of correctly classified cells was usual. Atypical cytomorphology in misclassified cells was observed in all cases. In the B/T-lymphoma group, misclassified neoplastic lymphocytes in MZL and HCL were medium-sized and had abundant basophilic or pale cytoplasm with projections and finer chromatin with nucleolus in contrast with correctly classified neoplastic cells. In MCL, atypical lymphocytes had a blastoid appearance in contrast with correctly classified neoplastic cells. Misclassification of neoplastic lymphocytes as blasts was also observed in a pleomorphic variant of CLL. In the AL+CMML group, misclassified lymphoblasts and myeloblasts were small, had a high nucleoplasmacytic ratio and had coarser chromatin with nucleoli. Numerous granular monoblasts were misclassified as promyelocytes. CMML cases were characterised by misclassifications involving dysplastic elements of the monocytic lineage. In the MM group, misclassifications occurred in the plasma cell lineage, especially in plasmablasts and immature plasma cells (Figure S5). Patients with critical misclassifications were exclusively in the diagnostic groups AL+CMML, MM and B/T-lymphoma. There were no patients with critical misclassifications in the MPN, MDN and reactive groups.

**3.4 The comparison of optical and digital myelograms**

***3.4.1 Overall results of the comparison***

The comparison was based on numerical differences in the representation of individual cell types between optical and ADM myelograms. Using these differences, the patient similarity network (PSN) was constructed to organise patients into a visually understandable form; for more details on PSN, see Methods. Based on the similarity, three separated subgroups of three diagnostic groups – AL+CMML (red), MM (blue) and B/T-lymphoma (orange) – were identified (Figure S6A), in which most patients with low values of relevant clinical consistency and all critical misclassifications occurred (Table S1; Figure S6B–C). The distribution of relevant clinical consistency values for each diagnostic group is shown in Figure S6C.


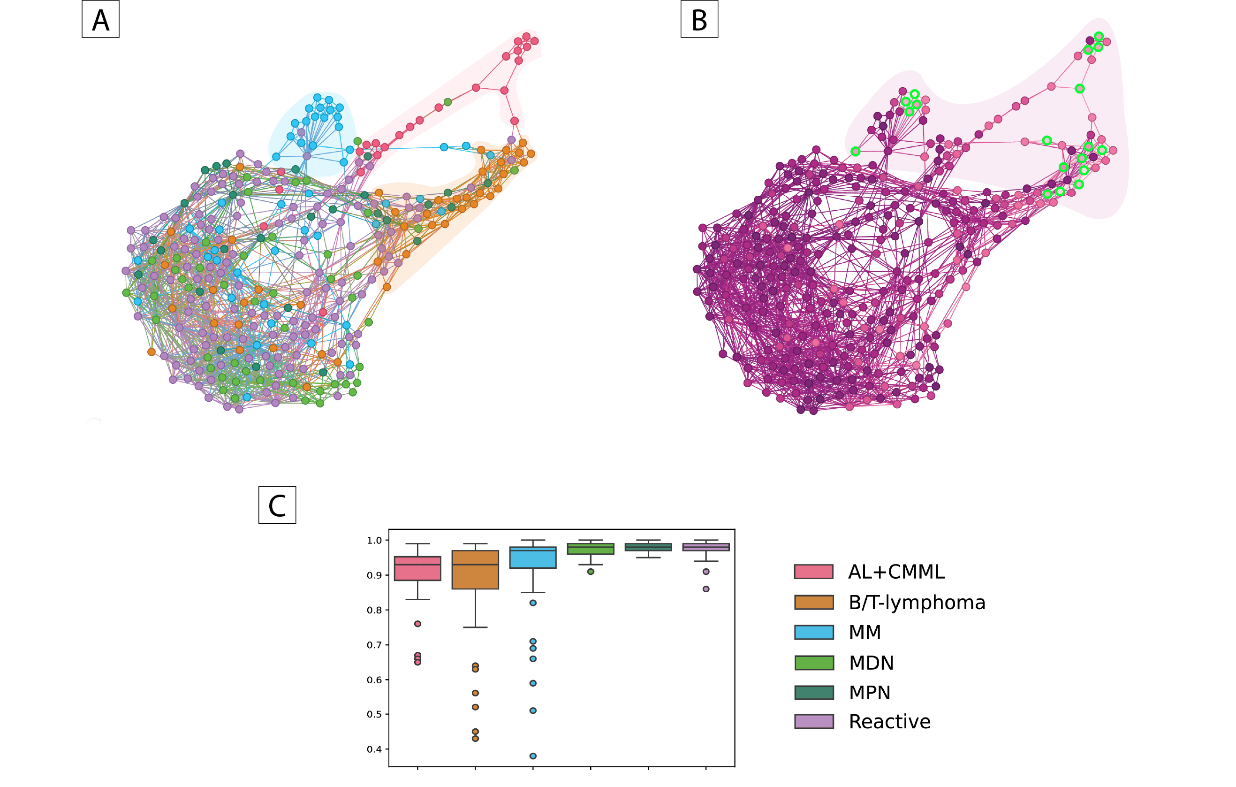


***Figure S6.*** ***The comparison of numerical differences between optical and digital myelograms and relevant clinical consistency across six diagnostic groups of haematological disorders. (A) Patient similarity network (PSN).*** First, we calculated numerical differences between expert and ADM evaluation for 25 cell types and selected the clinically relevant cell types. The PSN was constructed based on similarities of patients considering the differences between expert and ADM for the selected cell types. Each dot represents an individual patient; patients closer together share more similar myelogram discrepancies, whether they are lower or higher. The diagnostic groups of all cases are colour-coded. Three diagnostic subgroups of AL+CMML (red), MM (blue) and B/T-lymphoma (orange) groups were separated from the other patients in contrast to MDN (light green), MPN (dark green) and reactive (violet) groups. ***(B) Relevant clinical consistency values in the PSN.*** In the constructed PSN, the values of relevant clinical consistency are shown in purple. Low values of relevant clinical consistency are shown in light purple, and high values of relevant clinical consistency are shown in dark purple. Patients with critical misclassifications highlighted in green occur exclusively in the diagnostic groups AL+CMML, MM and B/T-lymphoma. *(****C)*** ***Display of relevant clinical consistency values in the diagnostic groups.*** The box plot view confirms the visualisation in the form of a network. Patients with low relevant clinical consistency occur predominantly in the diagnostic groups ALL+CMML, MM and B/T-lymphoma, including all patients with critical misclassifications.

***3.4.1 Differences in diagnostically critical cells***

Numbers of cases with an expert–ADM mismatch in particular cell types (dichotomised form, see Methods for details) in all diagnostic groups are shown in Table S2.


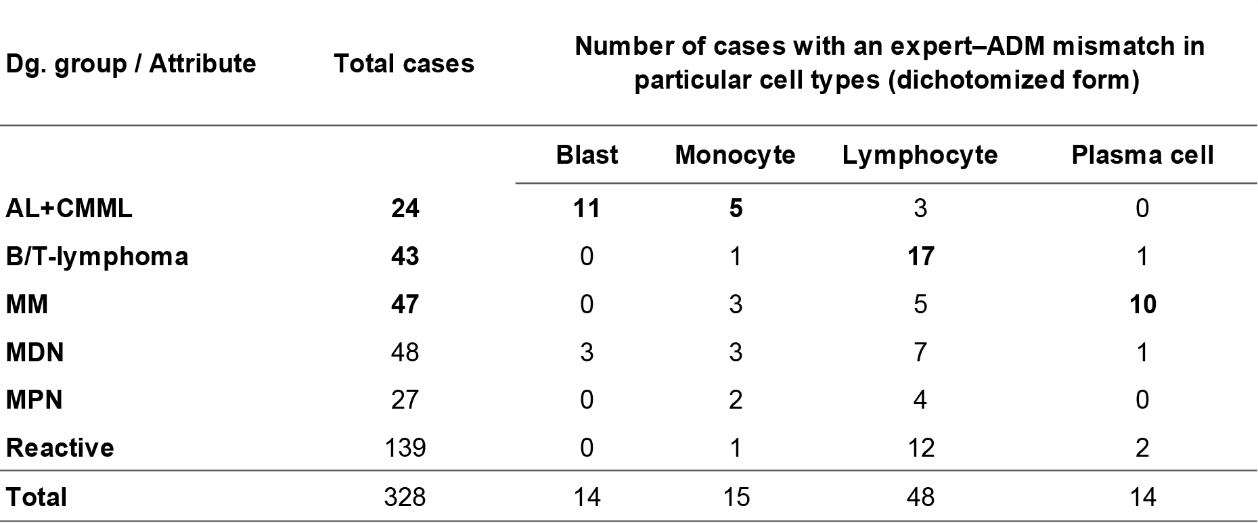


***Table S2. Occurrence of cases with an expert–ADM mismatch.*** For each of the six diagnostic groups (rows), the prevalence of high differences is shown, with a non-zero value for the corresponding scale (columns); the occurrences used for further analysis are in bold. These attributes have a non-negligible relative prevalence for the diagnosis in relation to the total number of cases.

Numerical differences in the representation of diagnostically critical cells (blasts including myeloblasts, monoblasts and lymphoblasts; monocytes; lymphocytes; and plasma cells including plasmablasts and immature plasma cells) were displayed in the network (Figure S7). The largest differences in blast counts occurred in AL+CMML, monocyte counts in AL+CMML, lymphocyte counts in B/T-lymphoma and plasma cell counts in MM, matching the original values and their scaling (Figure S7A–B). To quantify the association between these higher prevalences for the four attributes and the three diagnostic groups, we utilised two measures using dichotomised values. The ORs were calculated between the values of one attribute and one diagnosis in an unbalanced proportion to all other diagnoses (Figure S7C). However, we are aware the OR results should not be overestimated due to this imbalance and the relatively small number of occurrences. Therefore, we calculated the MCC for each attribute–diagnosis pair. The binary correlation (the predictability of an expert–ADM mismatch) was satisfactory only for the pair AL+CMML–blast and weak for the other three (AL+CMML–monocyte, B/T-lymphoma–lymphocyte and MM–plasma cell), with near-satisfactory correlation for MM–plasma cell. However, if we consider the visualisation, OR and MCC together for all the attribute–diagnosis pairs, the examined results can be considered satisfactory overall (Figure S7).

**
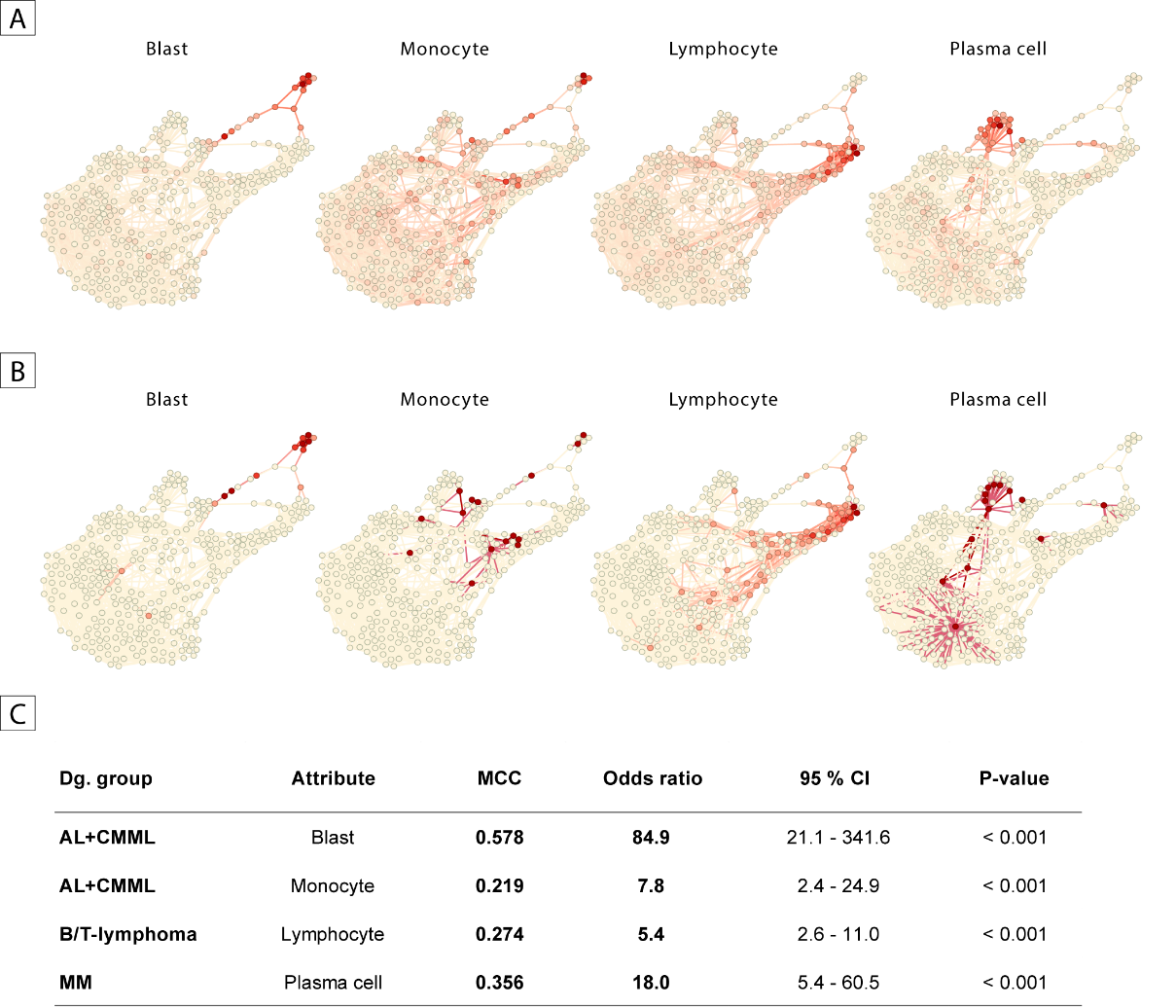
**

***Figure S7.*** ***The comparison of numerical differences of key attributes between optical and digital myelograms across six diagnostic groups of haematological disorders.*** *(*A, B) The key diagnostically relevant attributes (blast, monocyte, lymphocyte and plasma cell) were selected to show the numerical differences between the optical and ADM myelograms in the similarity network. The largest differences in the representation of blasts occurred in AL+CMML, monocytes in AL+CMML, lymphocytes in B/T-lymphoma and plasma cells in MM groups. This applies to both the original values (S7A) and their scaling (S7B). (C) MCC and OR ratio values were calculated for the key attributes in the diagnostic groups AL+CMML, MM and B/T-lymphoma, confirming the findings from the PSN.

**3.5 The diagnostic conformity of optical and ADM myelograms**

Diagnostic conformity was also assessed based on the relevance of the numerical ADM myelogram to a specific diagnosis. A total of 308 out of 328 cases showed diagnostic conformity between the ADM myelogram and the final diagnosis (overall, diagnostic concordance was 93.9%). In the group of 20 ADM discordant patients, 14 cases showed nonconformity of both optical and ADM numerical myelogram and only 6 cases showed only ADM diagnostic nonconformity (four patients with MM, one patient with AML and one patient with CMML). In the CMML patient, an ADM blast count above 20% classified the case as AML. In the AML patient, the ADM blast count was below 20%, classifying the case as MDN with increased blasts–2. In MM patients, the BM plasma cell count was below 10%.

***Discussion***

Prior studies conducted using modern automated instruments for PB smear assessments showed a strong correlation with manual assessment, with a pre-classification consistency ranging from 87% to 92%.^24–27^ However, there are limited data on the usefulness of ADM for BM assessment in haematology.

There is evidence of substantial inter-observer variability in manual BM smear analysis, which has downstream effects on patient diagnosis.^28, 29^ Due to the inherently subjective nature of conventional microscopic BM assessment, there is a pressing need for an automated approach to conducting standardised and objective BM cell differential counts.^24^ A recent Italian study demonstrated that expert evaluations of digitised BM aspirates provided fully comparable results to traditional microscopic analyses in routine diagnostics.^30^ However, the availability of standardised automation for this clinical diagnostic method remains limited.

The correct identification of individual cell morphological categories can be challenging, particularly in closely related classification categories with morphological similarities.^24, 27^ Expert findings, always influenced by a degree of subjectivity, may not be considered definitive or the only possible correct results – especially in cases of borderline or ambiguous cellular classification (e.g. in dysplastic or highly atypical elements).

Although ADM of BM is a promising modality with substantial potential to significantly improve diagnostic quality in haematology, reliable, comprehensive validation data for the Caucasian population have not yet been published. The available validation data from Asia, Israel, and the US display high consistency in cell classification, which is only concerned with cellular classification consistency.^5, 17–19, 31–33^

Therefore, our study aimed to comprehensively assess the effectiveness and reliability of the AI-assisted BM cytomorphology analysis system (Morphogo). In a real-world cohort of 328 BM smears from European patients, we focused on verifying the cellular and clinical classification consistency, the diagnostic validation of this method, and a comparison of numerical myelograms. In this study, we demonstrated the high image quality and classification capabilities of ADM and confirmed the excellent clinical consistency of ADM, with correct cell classification at 95.4% and clinical consistency reaching 97.1%. A strong classification correlation was observed in most cell categories, with absolute reliability in reactive haemopoiesis, MPN, and MDN.

Up to now, only a few studies have focused on ADM in BM diagnostics, especially with respect to cellular classification.^17–19, 31, 33^ In a recently published study comprising 508 BM cases categorised into five groups based on the degree of morphological abnormalities, using the Morphogo system, the classification consistency reached 99%, with most BM cell types showing an extremely high correlation with haematopathological expertise.^18^ Other promising comparisons of performance between automated PB and BM smear instrumentation and manual analysis were conducted in multicentre trials using the Scopio system.^19, 31, 33^ In the most recent of these, 795 BM specimens (615 Romanowsky-stained and 180 Prussian blue-stained) from patients with neoplastic and other clinical conditions were analysed, reaching an overall agreement of 91.1% with the reference method of optical microscopy.^19^ Our study showed similar classification agreement of optical microscopy and the Morphogo system.

The differences between our analysis and prior studies are mainly due to the varied representation of individual diagnostic categories, including rare diagnoses such as mantle cell lymphoma, hairy cell leukaemia, T-ALL or CMML, and the European ancestry of patients. In contrast to our cohort with 59% neoplastic conditions, in the Chinese study^18^, the proportion of haematological malignancies was less than 30%, and about one-fifth of the BM smears were classified as relatively normal.^19^ The representation of B/T-lymphomas in this study was 2.4%, in contrast to 13% in our cohort. Moreover, the methodology considering individual patients and numerical myelograms and using PSN to visualize differences between patients focused on clinical impact. Similar data from individuals of European ancestry have not yet been published. Future studies should be focused on the applicability of available ADM platforms in routine diagnostics.

This study was also the first to accurately identify the most frequently misclassified cell types with potential critical impacts on BM cytomorphological diagnosis. These included the misclassification of atypical lymphocytes resembling blasts, small lymphoblasts and myeloblasts resembling lymphocytes, granular monoblasts resembling promyelocytes, dysplastic elements of the monocytic lineage, plasmablasts, and immature plasma cells. Only three cell types – lymphoblasts, prolymphocytes and promonocytes – were systematically misclassified. In addition to cytomorphological overlap, this likely reflects the rarity of some diagnoses and inconsistency in expert consensus for certain cell types, resulting in incorrect annotations and gaps in training data. Thus, this study nominates candidate cells for future training and testing of AI capabilities.

Although these unfavourable outcomes are fully mitigated by expert supervision, we currently consider them a limitation of the absolute reliability of ADM. Mistaking lymphoma for AL and vice versa could have a fatal impact on patient management. False-negative cases, such as failure to recognise AML (especially acute promyelocytic leukaemia), or the misdiagnosis of CMML and MM, may result in medication misuse or inappropriate timing of treatment. Failure to treat these diseases could lead to patient death.

Similar misclassification patterns were reported for PB, showing stronger performance for common cell types but weaker classification consistency for some clinically important cell subsets, including blasts.^24–26^ The likely reason for the significant frequency of high-level misclassifications in our study is the different population-related distribution of some haematolymphoid neoplasms (especially mature B-cell neoplasms), which results in an incomplete AI training dataset. Another factor is the rarity of unique morphological pathologies. The composition of the study cohort – with the inclusion of rare types of B/T-lymphomas – highlighted challenges in recognising atypical lymphocytes by ADM. Similarly, detailed data on classification limits have not yet been published for any ADM system for BM analysis. In this regard, larger validation studies are essential, particularly in the Caucasian population.

In addition, we compared the numerical results of the myelogram from conventional analysis with those from expert ADM, as these are crucial for diagnostics in haematology. Surprisingly, the comparative analysis showed the largest differences in the representation of blasts in AL+CMML, monocytes in AL+CMML, lymphocytes in B/T-lymphoma, and plasma cells in MM. Although it is not entirely clear how to interpret this finding, it raises an important question regarding the accuracy of quantifying key diagnostic attributes, as well as a provocative question about the absolute correctness of expert opinion. Nevertheless, the majority of differences between the numerical myelogram results from conventional analysis and expert ADM were diagnostically neutral, with the exception of four cases of MM, one case of AML and one case of CMML.

One major reason for these numerical differences is the automatic selection of the adaptive area for 100× immersion lens analysis. This process is based on image segmentation, feature classification and evaluation, region scoring, score visualisation, avoidance of vacuole regions and evaluation of nucleated cell distribution uniformity. This elaborate and highly standardised method of selecting representative smear regions for analysis can, in many cases, be more reliable than region selection in conventional light microscopy, reducing variability associated with user bias in region and cell identification by analysing a significantly larger number of slide areas and cells.

The Morphogo system has shown a strong ability to identify various cell types, including megakaryocytes, and even metastatic cancer cells.^5, 17, 18, 32, 34–37^ Morphogo is also highly sensitive in detecting circulating plasma cells.^37^ AI-based ADM by Morphogo, including alternative categorisation proposals and cellular context display, represents an extraordinary and hitherto unexplored opportunity to facilitate BM cellular classification that can be valuable even to highly experienced morphologists.

Regarding future diagnostic utility of ADM in BM assessments, our study confirmed that ADM is helpful in routine clinical diagnostic use within the scope of its defined limitations. Implementing the method into routine diagnostics means replacing optical microscopy with digitization, recording of the required number of cells, subsequently reviewed by an experienced laboratory technician, a scientist or a physician and used as the basis for a diagnostic report. Despite a high degree of agreement between ADM and expert reclassification, expected in most cases, expert supervision is generally mandatory. A key advantage of ADM is the ability to analyse a large number of BM cells – up to 2,000 – in a short time, and image digitization enabling the remote expert review. In addition to enhancing the standardisation and objectivity of analysis, the practical integration of ADM into clinical laboratories will introduce beneficial workflow changes, reduce the burden on professionals, save time and provide remote access – ultimately translating into more affordable medical care. An implementation challenge lies in the standardisation of staining methods, image colour and size, and cell recognition. ^1,2,4,11,12^

Virtual slides do not deteriorate over time, have minimal storage requirements, are more accessible and available for reviews, consultations and publications along with the creation of digital image archives, extensive databases and libraries.^1,2,11^ The storage of digital data places considerable demands on server capacity, backup systems and cybersecurity.¹ ADM also enables significant improvement of morphology education, training and staff competency. In our experience, the training requirements and operational demands for staff are acceptable. Nevertheless, the high financial cost of acquiring the technology remains a barrier to broader implementation, which will be a long-term process.

Regarding the perspectives, further refinement of classification abilities and AI training focused on specific candidate cell types is essential to improving classification consistency, and the results of our study will support this aim. In the future, data and expertise from reference laboratories will contribute to the continued training of AI and the advancement of ADM. Within mature lymphoid neoplasms, an improvement of ADM could enable subclassification into specific diagnostic entities.^32^ ADM can also identify and analyse elements of megakaryopoiesis in BM and detect metastatic tumour cells.^35,36^ An AI-based approach to cytomorphology of megakaryocytes has the potential to significantly refine the integrative diagnosis of Ph-negative MPN.^34, 35, 38, 39^ A large margin also remains for reliable AI-supported recognition of lineage dysplastic changes.^1, 40–42^

This study has several limitations. First, the study included a limited number of patients, and some rare BM disorders were absent, including paediatric samples. Second, the cytomorphology of certain neoplastic cells was very abnormal, with questionable consensus in classification even among experienced experts; a second expert opinion was obtained for problematic cells to minimise misclassifications. Third, only samples of European ancestry were analysed. Fourth, no external validation of relevant and irrelevant misclassifications was performed; the experts were very experienced, regularly participating in successful external quality evaluations. Nevertheless, this exploratory study was the first to comprehensively identify critical points needing improvement in ADM classification capabilities. However, large validation datasets, including assessments of interpretative validity, are still lacking and need to be developed following this study.

In conclusion, the data demonstrate that ADM represents a fundamental paradigm shift for BM cytomorphology. The method used in this study has enormous potential to drive diagnostic transformation by reducing subjectivity and variability in assessments. It offers a novel and complementary perspective on BM cytomorphology, enhancing its quality and creating opportunities for clinical implementation. Nevertheless, a highly skilled morphologist will always be essential for definitive cell classification review and diagnostic interpretation, as expert assessment remains the cornerstone of morphological diagnosis in haemato-oncology. Expert-driven exploring the full potential of ADM in BM assessment will create opportunities to support routine laboratory work, especially in regions with a shortage of trained professionals.

1. ***References­***
2. Starostka D, Dolezilek R, Chasakova, K. Artificial Intelligence Increases Reliability in Diagnostic Hematooncology. *J Clin Transl Pathol.* 2023;3(3):146-147. doi: 10.14218/JCTP.2023.00015.
3. Zini G, Barbagallo O, Scavone F, Béné MC. Digital morphology in hematology diagnosis and education: The experience of the European LeukemiaNet WP10. *Int J Lab Hematol.* 2022;44(Suppl 1):37-44. doi: 10.1111/ijlh.13908. PMID: 36074713.
4. Xing Y, Liu X, Dai J, et al. Artificial intelligence of digital morphology analyzers improves the efficiency of manual leukocyte differentiation of peripheral blood. *BMC Med Inform Decis Mak* 2023;23(1):50. doi: 10.1186/s12911-023-02153-z. PMID: 36991420.
5. Kratz A, Lee SH, Zini G, Riedl JA, Hur M, Machin S. International Council for Standardization in Haematology. Digital morphology analyzers in hematology: ICSH review and recommendations. *Int J Lab Hematol.* 2019;41(4):437-447. doi: 10.1111/ijlh.13042. PMID: 31046197.
6. Jin H, Fu X, Cao X, et al. Developing and Preliminary Validating an Automatic Cell Classification System for Bone Marrow Smears: a Pilot Study. *J Med Syst* 2020;44(10):184. doi: 10.1007/s10916-020-01654-y. PMID: 32894360.
7. Tayebi RM, Mu Y, Dehkharghanian T, et al. Automated bone marrow cytology using deep learning to generate a histogram of cell types. *Commun Med (Lond).* 2022 Apr 20;2:45. doi: 10.1038/s43856-022-00107-6. PMID: 35603269; PMCID: PMC9053230.
8. Chumachenko K, Iosifdis A, Gabbouj M. Feedforward neural networks initialization based on discriminant learning. *Neural Netw.* 146, 220–229 (2022).
9. Ehteshami Bejnordi B, Veta M, Johannes van Diest P, et al. Diagnostic assessment of deep learning algorithms for detection of lymph node metastases in women with breast cancer. *JAMA* 318, 2199–2210 (2017).
10. Wu YY, Huang TC, Ye RH, et al. A hematologist-level deep learning algorithm (BMSNet) for assessing the morphologies of single nuclear balls in bone marrow smears: Algorithm development*. JMIR Med. Inform.* 8, e15963 (2020).
11. Esteva A, Kuprel B, Novoa RA, et al. Dermatologist-level classification of skin cancer with deep neural networks. *Nature* 542, 115–118 (2017).
12. Walter W, Haferlach C, Nadarajah N, et al. How artificial intelligence might disrupt diagnostics in hematology in the near future. *Oncogene* 2021;40(25):4271-4280. doi: 10.1038/s41388-021-01861-y. PMID: 34103684.
13. Lin E, Fuda F, Luu HS, Cox AM, Fang F, Feng J, Chen M. Digital pathology and artificial intelligence as the next chapter in diagnostic hematopathology. *Semin Diagn Pathol* 2023;40(2):88-94. doi: 10.1053/j.semdp.2023.02.001. PMID: 36801182.
14. Peng K, Peng Y, Liao H, et al. Automated bone marrow cell classification through dual attention gates dense neural networks. *J Cancer Res Clin Oncol* 149, 16971–16981 (2023). <https://doi.org/10.1007/s00432-023-05384-9>.
15. Gedefaw L, Liu CF, Ip RKL, et al. Artificial Intelligence-Assisted Diagnostic Cytology and Genomic Testing for Hematologic Disorders. *Cells.* 2023 Jun 30;12(13):1755. doi: 10.3390/cells12131755. PMID: 37443789; PMCID: PMC10340428.
16. Lee SH, Erber WN, Porwit A, Tomonaga M, Peterson LC. (2008) ICSH guidelines for the standardization of bone marrow specimens and reports. *Int J Lab Hematol.*, 30, 349–364.
17. WHO Editorial Board. WHO Classification of Tumours: Haematolymphoid Tumours, 5th Ed., World Health Organization, July 2024, ISBN: 978-92-832-4520-9.
18. Fu X, Fu M, Li Q et al. Morphogo: An Automatic Bone Marrow Cell Classification System on Digital Images Analyzed by Artificial Intelligence. *Acta Cytol* 2020;64(6):588-596. doi: 10.1159/000509524. PMID: 32721953.
19. Lv Z, Cao X, Jin X, Xu S, Deng H. High-accuracy morphological identification of bone marrow cells using deep learning-based Morphogo system. *Sci Rep.* 2023 Aug 17;13(1):13364. doi: 10.1038/s41598-023-40424-x. PMID: 37591969; PMCID: PMC10435561.
20. Bagg A, Raess PW, Rund D, et al. Performance Evaluation of a Novel Artificial Intelligence-Assisted Digital Microscopy System for the Routine Analysis of Bone Marrow Aspirates. *Mod Pathol.* 2024 Jun 17;37(9):100542. doi: 10.1016/j.modpat.2024.100542. Epub ahead of print. PMID: 38897451.
21. Chicco D, Jurman G. (2020). The advantages of the Matthews correlation coefficient (MCC) over F1 score and accuracy in binary classification evaluation. *BMC genomics*, 21, 1-13.
22. Janca O, Ochodkova E, Kriegova E, Horak P, Skacelova M, Kudelka M. (2023). Real-world data in rheumatoid arthritis: patient similarity networks as a tool for clinical evaluation of disease activity. *Applied Network Science*, 8(1), 57.
23. Trajerova M, Kriegova E, Mikulkova Z, Savara J, Kudelka M, Gallo J. Knee osteoarthritis phenotypes based on synovial fluid immune cells correlate with clinical outcome trajectories. *Osteoarthritis Cartilage.* 2022 Dec;30(12):1583-1592. doi: 10.1016/j.joca.2022.08.019. Epub 2022 Sep 17. PMID: 36126821.
24. Ochodkova E, Zehnalova S., Kudelka M. Graph construction based on local representativeness. In: *International Computing and Combinatorics Conference.* Springer. 2017, pp. 654–665.
25. Lewis JE, Pozdnyakova O. Digital assessment of peripheral blood and bone marrow aspirate smears. *Int J Lab Hematol.* 2023 Jun;45 Suppl 2:50-58. doi: 10.1111/ijlh.14082. Epub 2023 May 21. PMID: 37211430.
26. Briggs C, Longair I, Slavik M, et al. Can automated blood film analysis replace the manual differential? An evaluation of the CellaVision DM96 automated image analysis system. *Int J Lab Hematol.* 2009; 31(1):48-60.
27. Ceelie H, Dinkelaar RB, van Gelder W. Examination of peripheral blood films using automated microscopy; evaluation of Diffmaster Octavia and Cellavision DM96. *J Clin Pathol.* 2007;60(1):72-79.
28. La Gioia A, Fiorini F, Fumi M, et al. A prolonged microscopic observation improves detection of underpopulated cells in peripheral blood smears. *Ann Hematol.* 2017;96(10):1749-1754.
29. Sasada K, Yamamoto N, Masuda M, et al. Inter-observer variance and the need for standardization in the morphological classification of myelodysplastic syndrome. *Leuk Res.* 2018;69: 54-59.
30. Naqvi K, Jabbour E, Bueso-Ramos C. Implications of discrepancy in morphologic diagnosis of myelodysplastic syndrome between referral and tertiary care centers. *Blood.* 2011;118(17): 4690-4693.
31. Zini G, Chiusolo P, Rossi E, et al. Digital morphology compared to the optical microscope: A validation study on reporting bone marrow aspirates. *Int J Lab Hematol*. 2024; 1-7. doi:[10.1111/ijlh.14238](https://doi.org/10.1111/ijlh.14238)
32. Bagg A, Raess PW, Rund D, et al. Performance evaluation study of a novel digital microscopy system for the quantitative analysis of bone marrow aspirates. *Blood.* 2021;138: 4000.
33. Tang G, Fu X, Wang Z, Chen M. A Machine Learning Tool Using Digital Microscopy (Morphogo) for the Identification of Abnormal Lymphocytes in the Bone Marrow. *Acta Cytol* 2021;65(4):354-357. doi: 10.1159/000518382. PMID: 34350848.
34. Katz BZ, Feldman MD, Tessema M, et al. Evaluation of Scopio Labs X100 Full Field PBS: The first high-resolution full field viewing of peripheral blood specimens combined with artificial intelligence-based morphological analysis. *Int J Lab Hematol.* 2021;00:1–9.
35. Sirinukunwattana K, Aberdeen A, Theissen H, et al. Artificial intelligence-based morphological fingerprinting of megakaryocytes: a new tool for assessing disease in MPN patients. *Blood Adv.* 2020 Jul 28;4(14):3284-3294. doi: 10.1182/bloodadvances.2020002230. PMID: 32706893; PMCID: PMC7391156.
36. Wang X, Wang Y, Qi C, et al. The Application of Morphogo in the Detection of Megakaryocytes from Bone Marrow Digital Images with Convolutional Neural Networks. *Technol Cancer Res Treat.* 2023 Jan-Dec;22:15330338221150069. doi: 10.1177/15330338221150069. PMID: 36700246; PMCID: PMC9896096.
37. Chen P, Chen Xu R, Chen N, et al. Detection of Metastatic Tumor Cells in the Bone Marrow Aspirate Smears by Artificial Intelligence (AI)- Based *Morphogo* System. *Front Oncol.* 2021 Sep 27;11:742395. doi: 10.3389/fonc.2021.742395. PMID: 34646779; PMCID: PMC8503678.
38. Chen P, Zhang L, Cao X, et al. Detection of circulating plasma cells in peripheral blood using deep learning-based morphological analysis. *Cancer.* 2024 May 15;130(10):1884-1893. doi: 10.1002/cncr.35202. Epub 2024 Jan 18. PMID: 38236717.
39. Ryou H, Lomas O, Theissen H, Thomas E, Rittscher J, Royston D. Quantitative interpretation of bone marrow biopsies in MPN-What's the point in a molecular age? *Br J Haematol.* 2023 Nov;203(4):523-535. doi: 10.1111/bjh.19154. Epub 2023 Oct 19. PMID: 37858962.
40. Belcic T, Cernelc P, Sever M. Artificial intelligence aiding in diagnosis of JAK2 V617F negative patients with WHO defined essential thrombocythemia. *HemaSphere.* 2019;3(S1):998.
41. Kimura K, Tabe Y, Ai T, et al. A novel automated image analysis system using deep convolutional neural networks can assist to differentiate MDS and AA. *Sci Rep.* 2019;9(1):13385
42. Brück OE, Lallukka-Brück SE, Hohtari HR, et al. Machine learning of bone marrow histopathology identifies genetic and clinical determinants in patients with MDS. *Blood Cancer Discov.* 2021;2(3):238–49.
43. Haferlach T, Pohlcamp Ch, Heo I, et al. Automated peripheral blood cell differentiation using artificial intelligence—a study with more than 10,000 routine samples in a Specialized Leukemia Laboratory. *Blood.* 2021;138(Suppl. 1):103. https://doi.org/10.1182/blood-2021-152447
